# Furrowed Enamel in Connection with Syphilitic and Other Exanthematous Diseases

**Published:** 1873-06

**Authors:** S. P. Cutler

**Affiliations:** Memphis, Tennessee.


### SELECTED ARTICLES.

---

### ARTICLE III.

### *Furrowed Enamel in Connection with Syphilitic and other Exanthematous Diseases.*

BY S. P. CUTLER, M. D., MEMPHIS, TENNESSEE.

*Case* 1.—A lad, about 17 years of age, under size, Scottish parentage and birth, has rather a scrofulous appearance, sallow complexion.

The six front teeth, above and below, are all deeply grooved near the cutting edges, three grooves across each on front side, with intervening deep pits, with marked uniformity in all, there being scarcely any difference in the order of grooving and pitting; the cutting edges in all deeply notched, narrowed, and contracted. The teeth, between the grooves and gums, are perfectly developed, smooth, and sound; none of the pits or grooves decayed. The sixteen teeth, back of the twelve spoken of, are well developed, and sound, except one upper 6 year molar, which was badly decayed, and had to be removed. This was owing to the fact that these teeth were not yet far enough advanced to suffer from the dermal disease which I am about to describe, which will equally apply to the other cases mentioned, and all other similar cases, whether the effect of the one or the other varieties of exanthematous diseases, discussed in this paper.

The father of the boy has a similar sallow look as the boy, though stout and healthy, only subject to bilious disturbances. The mother is a stout healthy woman, and the mother of a large family of healthy children. The temperaments of both father and son are sanguine bilious.

The following is the history of this singular case:

The boy, up to three months old, was perfectly healthy and robust. At that age, the child was vaccinated with spurious or bad matter, which came from London, the family residing in Glasgow, Scotland. At first the sore progressed favorably, though phagidenous. As the sore began to heal, a breaking out, all over the body, began to make its appearance, which, from the description, resembled secondary syphilis. The doctor, supposing the matter to be fresh from the cow, had his suspicions excited concerning its genuineness, and at once put the child under treatment, though a long time elapsed before the eruptions disappeared; the child becoming, and continuing sickly, until they started to America, the patient being then nearly three years old, and without any signs of teeth in his jaws. After they had been on shipboard about one week, the child began to want to eat everything he saw, after which time he began to cut teeth. The father did not recollect whether there was anything peculiar about the first set of teeth, only that they were defective. It is but reasonable to conclude that the first set were defective.

This is an interesting case, and would lead to the conclusion that the vaccine matter must have been taken from a syphilitic subject, as there never had been a case of syphilis known in any of the ancestry on either side. The physician at Glasgow believed it to be a mixed case; he did not use any more of the vaccine matter, nor any of the matter from the child's arm, as he had intended to do. This, then, might be regarded as a mixed case, of vaccine and syphilitic poisoning, combined; the vaccine disease running its usual course, the sore being chancrous, subsiding with secondary syphilis, which was ultimately cured, by the treatment while in Scotland.

I believe it is a well established pathological fact that no two acute diseases can run their course, in the human organism, at the same time, or together; the weaker one must lie dormant until the more energetic one has run its course; then the other may run its course, unless the cause of the one be overcome by the energy and activity of the other. There may be cases where two acute diseases, of a specific character, may run their specific courses in succession, as in the case under consideration, the one waiting for the other to run its course. In the above cited case, one disease took on its only form, the acute, while the other took on the secondary; as a sequel, if I may so express it, to the acute form of the other.

It is now, I believe, generally admitted, that when secondary syphilis is inoculated into the system of a second person, that the disease manifests itself only in the secondary form, passing entirely over any acute or initiatory symptoms or tage—not even producing true chancre. The above cases did simulate true chancre, though a mixed case, neither, perhaps, being absolutely genuine, the protecting power of the vaccine being questionable,

Does not the above case point out, clearly, the close and intimate relation between the enamel of the teeth and epidermis, and inferentially, that of the dermis, or true skin, and the dentine ? The two former being exuvious in character, the enamel being retained in consequence of its hardness, both being vital and non-vital, desquamation of epidermis not taking place so long as there is any vital connection remaining. The scaly eruptions over the body of the child continued over twelve months, and under treatment during the whole time, which accounts for the non-appearance of teeth up to the third year, which is a very extraordinary occurrence, and distinctly points out the dermic connection with the dental tissues.

This case more fully convinces me that my former views, already published, were correct—that is, that the teeth embody, or represent, with themselves, the three great divisions of the animal economy—the neuro-dernal, and the dermal ; the pulp, with its nerves and blood-vessels, direct from two of the three great vital centres, the brain and heart, the lungs forming the third vital centre, not represented in the structure of the dental organs.

Extensive capillary blood-vessels, veins, and arteries, as well as nerve-fibrils and plexuses, ramify extensively through the skin, similar to the dental pulp, the arrangement of the nerve fibrils, in the latter, forming, to some extent, an exception, as they project through the pulp into the dentinal tubuli, up to the enamel, and cementum, or crusta-petrosa, the blood-vessels stopping at pulp boundaries.

In the above case, the twelve teeth have thin edges where the plates of enamel met, from opposite sides, are defective in development at those points. The balance of the teeth, having broader grinding or cutting surfaces, where the enamel is much less in proportion to the dentine, were not at all affected by the disease. There was an arrest of enamel development, from want of germinal matter, or nutrition, in the enamel's basement membrane, or *primus formatibus*, of the twelve teeth named, or at least a want of normal ossification, at the points of those teeth. I have assumed that there was a want of proper nutrition during the developmental stages of these teeth. In saying this much, I do not assume it as a *dictum*, only an hypothesis, with good reasons to sustain my position.

We have in the above a clear case of neuro-dermal resistance in the dentine, of the sixteen teeth not affected by the taint, and portions of the others, to the influence of the disease. Only where the proportion of the enamel to dentine is greatest, is there any defective development.

Scarlatina, measles, and other varieties of exanthemous diseases, are known to produce similar conditions of the teeth, at certain ages, in favorable temperaments, while others will be wholly exempt from any such sequela. The same may also be said of inherited syphilis; some children may inherit the disease, while others will be wholly exempt from any taint whatever, apparently.

I saw a case the other day, a lad thirteen years old, who had measles at the age of three years, with the four six-year permanent molars, on their grinding surfaces, deeply pitted, with sharp points studding the entire cutting surfaces, the borders of which were grooved and narrowed, the defects being confined to the enamel alone; all the other teeth were natural, except the upper central incisors, which had a broad notch in the middle, or cutting edge, of each; teeth all sound and well developed, though the jaws were not in proportion, being narrowed, causing some irregularity and crowding at the site of the canines. There were no indications of any syphilitic taint, in this case, or even suspicions. Published observations, on the part of medical men, are very meagre, and hospital reports, up to the present time, are not sufficiently extensive to base conclusions upon, on this subject.

It is well known that all exanthematous diseases are of a specific character; though all belonging to the same family of diseases, neither one is protective against either of the others, except that of vaccine, which, is a modification of variola, brought about by the modifying effects of vaccination.

None of the other forms of exanthema are susceptible of modifying exemptions, except immunity from subsequent attacks, as a rule, to which, like all other general rules, there are exceptions.

Syphilis, so far as known, is received into the system by inoculation only, except by hereditary taint, from parents, So far as is known, this disease is never contracted through the lungs, by contaminated atmosphere. This disease is, at first, only a local sore, of a specific character, remaining local for an indefinite period, varying in different individuals, owing to idiosyncrasies, being carried through the system by absorption of the local virus, which is cumulative in character—*a true ferment*, capable of re-developing itself, as it pervades the tissues, appropriating to itself the elements for regeneration; hence, such tissues suffer most by its ravages.

The stage of local probation may vary in different subjects, owing to modifying circumstances, the local inflammation gradually extending itself through the system, contaminating cell after cell, as it travels, until the more susceptible tissues are more or less completely saturated with the virus.

This saturation results in febrile reaction, of longer or shorter duration. After running its febrile course, it then subsides into the secondary, and, ultimately, the tertiary, or more thoroughly constitutional form, the more readily transmissible to offspring, and becomes a fixture, by producing certain modifications of cells, of different tissues, some more than others, which may, or may not, be entirely curable in every case.

To what extent the secondary form of the disease may modify a subsequent attack, has not been clearly defined yet, by authors. That the same subject may have repeated attacks I am satisfied; though there is, at least in some cases, marked modifications of the disease, in subsequent attacks, by a species of *acclimation*, (if the term is allowable)—an immunity from severity, or, in some cases, altogether exempted, similar to other infectious or contagious diseases.

All the exanthematic forms of disease are, I believe, regarded as zymotic, or fermentative; of fermentative elements, in the fluids and solids of the body, consisting of the tertiary group of compound proximate principles; the azotized principles, constituting the elements of the ferment, or yeast, which plays a catalytic career only in the process. This fermentation, after pervading the whole organism, fluids and solids, capable of coming under this chemical influence, in opposition to vital resistance, destroys, more or less completely, the susceptibility of any similar process of the exact nature and origin of the first, notwithstanding the constant change of the entire organism, by constant waste and reproduction, this modifying influence being retained, more or less perfect, perhaps through life.

Syphilis, unlike any other of the zymotic tribe of diseases, has its acute stage, definite in its character; also a sub-acute stage, definite in character, following the acute, unless arrested by suitable treatment, by complete eradication. This sub-acute stage has limits, which probably subsides into the tertiary, or permanent constitutional variety, of the disease, becoming hereditary, or transmissible to offspring.

Each stage of the same disease is specific in character, and, if transmitted, that distinct form of the disease is transmitted only; if from the primary chancre, the three stages, in succession, may supervene; not so when the disease is transmitted from the secondary stage, by inoculation—the genuine primary chancre, and acute local and general symptoms are not developed, only the secondary variety; the third stage being transmissible alone by hereditary descent, from the best light and knowledge before us.

None of the other forms of zymotic exanthema ever become constitutional, and transmissible by hereditary descent.

None of them have any distinct secondary type, only a sequala of the acute stage, not even contagious during this stage, if so at all; the acute disease of the genuine type, alone, could be produced, excepting, as before stated, the modified form of variola, by vaccination, or varioloid.

Mr. Hntchinson, in the *London Hospital Reports*, vol. ii. p. 56, shows, from actual personal observation, that tertiary syphilis is transmissible by descent, after the parent had been apparently entirely cured for a number of years, and that such offspring frequently manifest such evidence, by their notched, grooved, and otherwise defective dental developments. In some instances given, the bones of the nose alone give evidence of the taint, by flattening, and want of development, on others, interstitial keratitis is observed, as a constitutional taint. Many other characteristic distinctives might be named, furthering these ideas.

Lancereaux and others maintain that constitutional or tertiary syphilis degenerates the man into something inferior, and no longer a man, having undergone a systemic change throughout.

Hunter, in 1786, first proved the specific nature of venereal disease, by inoculation, which was more recently confirmed by Ricord.

In 1813, Mr. Carmichael first treated these diseases without mercury, or the first to bring it to public notice; he used mercury when other treatment failed.

Prof. Gross, in his *Surgery*, says: "It is probable that the system, when once affected, nearly always retains the impress, so as to be transmissible from parent to offspring, during a series of generations." He further says: "The method of treatment has an important influence in etablishing a constitutional diathesis, especially mercury, in the first stage of chancre."

Parker, in his book on *Venereal*, says: "It is not capable of transmission by hereditary descent, but by inoculation of fœtus, at birth." And again: "Tertiary symptoms may make their appearance at long intervals, after the primary have disappeared, or during the existence of the secondary, or even long after their subsidance, as patches on the skin, when the affection is of the tertiary form."

Sometimes this variety succeeds that of the chancre, without any affections of the skin or throat. This form may communicate, by contact, and produce the same form of disease, *per se.*

Ricord says: "When the chancre is cured in five days, no secondary symptoms follow." No recorded instance.

M. Cazenave, Erasmus Wilson, and Dr. Egan, have seen constitutional diseases without any chancre, only a discharge from urethra. They say inoculation does not produce constitutional disease in the same individual.

Waller, and Vidal de Cassis, have produced the disease in the healthy.

M. Boudeville, *Interne in Pharmacis*, says: "Inoculation produced the disease, in the secondary form, same as the patient from whom the matter was taken."

This author thinks that, when the father alone is affected, the secretions, coming in contact with the ovum, causes the taint, and not from physiological causes. Thinks it is, when the mother has secondary disease, in every instance.

In 153 cases, 46 had taken mercury for primary disease, and 76 none.—*Quin's Hospital Reports*, *Birmingham.*

Ricord's experiments show that malignant bubo, by inoculation, will produce the same sore.

Dr. Wallace, of Dublin, succeeded only three times in many hundred experiments.

Prof. Gross says: "Tertiary syphilis sometimes follows secondary, when this form has deeply penetrated the system, by gradual transition from one into the other, sometimes supervening in the course of five or six months, sometimes not until eighteen years have transpired; then coming on suddenly. In some cases no secondary symptoms supervene, the primary ending in the tertiary form. The matters from tertiary ulcers and sores are not inoculable, and not transmissible into specific form, though in some other constitutional manner."

In his *Surgery*, he seems to have paid no attention to hereditary or induced affections of children's teeth, not making any mention of the fact, of which we have abundant evidence in numerous cases cited.

The difference in specific characteristics of hereditary taints, and that contracted *in parturio*, must necessarily be considerable, in general symptoms, though perhaps similar in dental deformities, when such take place, varying chiefly in degree. Here is where we need more light on this important subject, so intimately connected with the welfare of so many unfortunates.

Syphilization, spoken of, is not of sufficient importance to require notice here, from the fact that it has not been, nor will it ever be, resorted to, except experimentally.

Prof. Gross further believes that secondary syphilis is not inoculable, or contagious, from the fact that the virus undergoes modifications after entering the system, notwithstanding the experiments of Vidal; Cazenave and others still believe it transmissible from parent to offspring, declaring itself in a great variety of affections; sometimes proving destructive to the new offspring before or after birth. He believes that when once affected, every globule of blood, and every particle of solid matter, is impressed by the poison.

The premonitory and culminating symptoms are well defined, followed by the surface culminations.

The blood, in the acute stage, does not show any change in corpuscles. In advanced cases, profound alterations take place in all the secretions of the body, as well as solids, in many constitutions.

Ricord thinks that tertiary syphilis is not hereditary, but that the offspring may be scrofulous or cancerous, and this may be owing to the bad condition of the blood, secretions, and tissues.

*Microscopic Condition of the Blood in Secondary Syphilis.*—"The corpuscles are smaller in quantity, irregular in shape, small in size, ragged edges, coalescing and adhering. In the advanced stage, the globules are converted into albumen."—*M. Grasse.*

2

M. Waller succeeded in inoculating with the blood of advanced cases; also MM. Diduy and Vidal de Cassis believe in its contagious nature.

Globules of blood, after a few days, run together, and lose their form, on a glass slide, and show only a red stain.

The latest, and perhaps the most advanced views, on the subject of the disease, have been advanced by Lionel S. Beale. Under the head of *Diseased Germs*, he says: "This is another of those remarkable special living poisons, which may be suspended in serum and other fluids, and retain its vitality for any length of time."

There is reason for thinking that a single epithelial cell may carry multitudes of active particles of syphilitic poison, one of which, introduced in the blood or lymph of a healthy person, would probably grow, and multiply, and give rise to pathological changes, characteristic of, and quite peculiar to, this particular poison.

Again Dr. Beale says: "We know that syphilitic poison may retain its specific characteristics in the organism for years, from time to time giving rise to local pathological phenomena, which are characteristic of this kind of morbid bioplasm."

It is impossible, from the facts of the case, to arrive at any other conclusion than this, that a certain portion of the living matter remains in the organism, and that under certain favorable circumstances, this grows, and multiplies, producing disease. Particles of this virulent poison may be transferred from the affected organism to a healthy one, and contaminate it, even many years after its introduction into the first had taken place.

Of syphilitic bioplasm there are different kinds, giving rise to different pathological affections belonging to the syphilitic class. Indeed, some facts render it probable that there are several different species, or varieties, of syphilitic poison, instead of only one or two.

One very remarkable property of this poison is, that it may be inoculated into the same organism over and over again, until inoculation ceases to produce any effect. As soon as this is the case, it is said the organism is protected. But such protection, sometimes, cannot be produced, until successive inoculation has been practiced during several months, and, as has been remarked, the remedy is worse than the acquired disease, besides being, on many grounds, quite unjustifiable.

*To be Continued.*

